# Power analysis for conditional indirect effects: A tutorial for conducting Monte Carlo simulations with categorical exogenous variables

**DOI:** 10.3758/s13428-022-01996-0

**Published:** 2022-11-28

**Authors:** Samuel Donnelly, Terrence D. Jorgensen, Cort W. Rudolph

**Affiliations:** 1 Department of Psychology, Saint Louis University, 3700 Lindell Blvd, St. Louis, MO 63103 USA; 2 Graduate School of Child Development and Education, University of Amsterdam, Amsterdam, Netherlands

**Keywords:** Moderation, Mediation, Moderated mediation, Monte Carlo simulation, Power analysis

## Abstract

Conceptual and statistical models that include conditional indirect effects (i.e., so-called “moderated mediation” models) are increasingly popular in the behavioral sciences. Although there is ample guidance in the literature for how to specify and test such models, there is scant advice regarding how to best design studies for such purposes, and this especially includes techniques for sample size planning (i.e., “power analysis”). In this paper, we discuss challenges in sample size planning for moderated mediation models and offer a tutorial for conducting Monte Carlo simulations in the specific case where one has categorical exogenous variables. Such a scenario is commonly faced when one is considering testing conditional indirect effects in experimental research, wherein the (assumed) predictor and moderator variables are manipulated factors and the (assumed) mediator and outcome variables are observed/measured variables. To support this effort, we offer example data and reproducible R code that constitutes a “toolkit” to make up for limitations in other software and aid researchers in the design of research to test moderated mediation models.

## Introduction

This paper demonstrates how to conduct Monte Carlo power analyses (Muthen & Muthen, 2002) for tests of (moderated) mediation using the R package simsem (Pornprasertmanit et al., 2021). While similar tutorials already exist (e.g., Schoemann et al., 2014), our scope extends these methods by considering previously ignored aspects of sampling designs: first, the inclusion of fixed covariates, as in the case of experimental^1^ designs; second, the flexibility of multigroup SEM to model moderation of indirect effects. We begin with a brief comparison of established methods for estimating statistical power, noting the current gaps that our tutorial is designed to fill.

Statistical power defined under the frequentist logic of null hypothesis testing is the probability of detecting a significant effect in a sample if that effect in fact exists in the population. Power is equal to 1 − β (β being the probability of a type II error), or the probability of correctly rejecting the null hypothesis (Cohen, 1988). Statistical power has a relationship with sample size, effect size, and alpha (α), where α is the probability of making a type I error, or incorrectly rejecting the null hypothesis (i.e., one’s “significance level”). In this relationship, power increases as sample size, effect size, or α increases. In the case of moderated mediation, power is estimated specifically for the difference between indirect effects across levels of the moderator, also known as an equivalence test of indirect effects (MacKinnon, 2008).

Often researchers are interested in estimating the required sample size for obtaining the smallest effect size of interest (SESOI), which is the smallest effect size one would consider meaningful (e.g., Anvari & Lakens, 2021; Lakens et al., 2018). Too small of a sample size may result in making a type II error (which is debilitating to scientific progression), whereas too large of a sample may unnecessarily consume valuable resources (e.g., money to compensate unneeded participants, participant’s time in completing study procedures). Therefore, being prudent and properly conducting an a priori power analysis should very much be of interest to researchers.

There are analytic approaches to estimate power using general linear models (GLM; e.g., G*Power; Faul et al., 2007), based on functions of (specified or estimated) power, sample size, H_0_ test criterion, and standard effect size. There are also analytic approaches for structural equation models (SEMs) such as likelihood ratio test (e.g., LRT; Satorra & Saris, 1985) and root mean square error of approximation (e.g., RMSEA; MacCullum et al., 1996) that are capable of power analysis for more complex multivariate models. However, these methods are generally limited to ideal data (i.e., normally distributed, complete observations) and have yet to be extended to common real-data scenarios (e.g., discrete indicators, incomplete data). A more recently adopted method, Monte Carlo (MC) simulation, is more flexible and resolves many of the limitations noted above by estimating power of various test statistics (e.g., normal-theory-based *t* and *F* statistics in GLM, asymptotic *z* and χ^2^ statistics in SEM). MC-based power analyses also enable estimating power of more flexible (e.g., resampling) methods of testing a H_0_, such as percentile-based bootstrap confidence intervals, which can have differential power from analytically derived test statistics (Fossum & Montoya, 2021). MC is a resampling-based method which simulates data from a specified population model with parameters selected by the researcher (potentially derived from estimated parameters using pilot data). Empirical estimates of power to reject the null hypothesis (H_0_: effects = 0 in the population) under the specified effect sizes and sample size are calculated from the proportion of samples found to be significant at a given criterion (e.g., *p* < .05). For instance, if 10,000 simulated samples were taken and the effect of interest (e.g., *ab*) was found to be statistically significant for 8000 simulated samples, then the empirically estimated power to reject H_0_ would equal 80%.

Despite advances in the use of MC power analysis in the literature, specific gaps remain. Below we describe these gaps and illustrate our motivation for this tutorial. Generating data from a population model typically involves multivariate normal data generated from the model-implied mean and covariance matrix. These may be discretized with a threshold model, or missing-data mechanisms may be imposed. However, popular software facilitating MC simulations for SEM (e.g., M*plus*; Muthén and Muthén, 2002) generally do not provide a way to simulate different distributions of exogenous predictors, making it difficult to design a MC study for power analysis that accurately reflects the real data-generating process a researcher expects to encounter. For instance, age or income distributions might be determined by the study design, or more complex stratified sampling might be involved (Kroese et al., 2011, ch. 9). Incorporating fixed covariates falls under a larger set of variance-reduction techniques for Monte Carlo research (Dagpunar, 2007, ch. 5) that, in the context of power analysis can provide more stable estimates of power (Mayer & Thoemmes, 2019).

The general lack of user-friendly software capable of accepting a set of fixed covariates to be used for data-generation—in conjunction with the absence of explanatory literature on conducting MC simulation power analyses for conditional indirect effect models with categorical exogenous variables—has left researchers with a discernible methodological deficiency. An urgency to address this gap is indicated by the advocation of such models for experimental research (e.g., Lench et al., 2014), empirical investigations (e.g., Rudolph et al., 2015; Welsh et al., 2020) and numerous online inquiries^2^ (e.g., Research Gate, lavaan forum, WebPower). As far as we know, only the package simsem (Pornprasertmanit et al., 2021) in the open-source programming language R (R Core Team, 2021) can facilitate user-friendly^3^ simulations of SEM data that contain fixed exogenous predictors with arbitrary distributions. This tutorial seeks to comprehensively demonstrate how MC power analysis works in the case of moderated mediation in experimental designs, although in principle the same feature can be exploited for other cases mentioned above (e.g., fixed age or income distributions in a common-factor model).

In pursuit of this objective, we first briefly introduce moderated, mediated, and moderated mediation with categorical exogenous variables, as well as single-group and multigroup model approaches, each of which are employed throughout the tutorial. For both approaches, we begin the demonstration with a simple mediation model and then extend it to conditional indirect effect models. Lastly, we briefly note how to test and estimate power for (conditional) indirect effects using two^4^ methods: (1) Wald tests based on delta-method *SE*s (which should suffice in large samples), and (2) a parametric bootstrap technique referred to as Monte Carlo confidence intervals (MCCI), which are more robust in smaller samples (Preacher & Selig, 2012) and provide a less computationally intensive alternative to nonparametric bootstrap with similar results (Fossum & Montoya, 2021; Hayes & Scharkow, 2013).

### Model conceptualizations and approaches

**Fig. 1:**
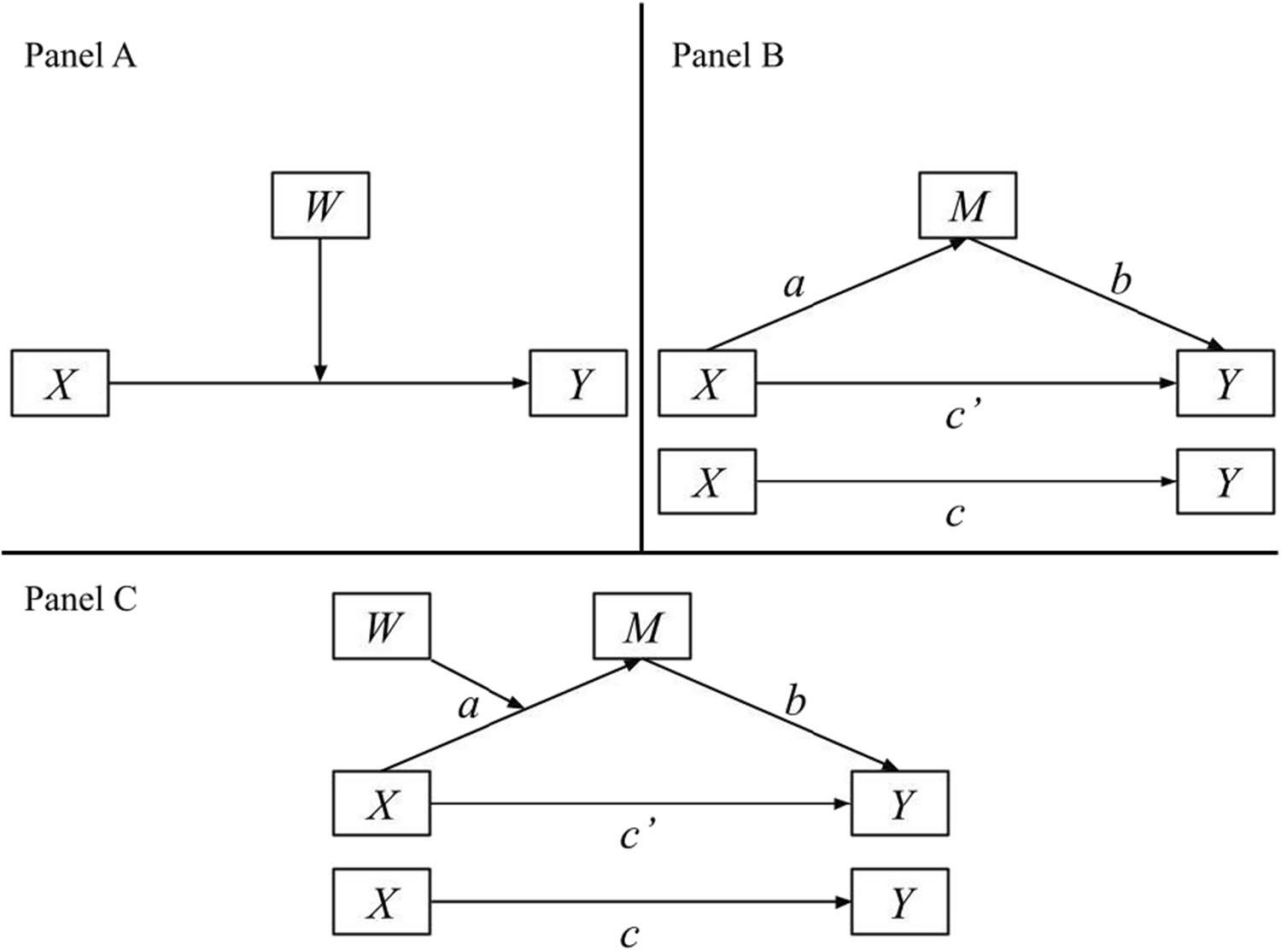
Illustrative model examples. *Note*. Panel A depicts a common case of moderation, Panel B depicts a common case of mediation, and Panel C depicts a common case of moderated mediation

Testing theories and hypotheses proposing moderated and mediated relations have become increasingly common throughout behavioral research. Moderation (see Fig. 1A) is commonly modeled as a statistical interaction effect by using the product of a focal predictor and moderator as an additional covariate and is broadly said to occur when the effect (i.e., strength and/or direction) of a focal predictor *X* on an outcome variable *Y* depends on the level of another variable *W* (moderator). In the simplest case, mediation (i.e., an indirect effect, see Fig. 1B) can be described as an independent variable *X* affecting an outcome variable *Y* through a third variable *M* (mediator). That is, the independent variable *X* affects the mediator *M*, which in turn affects the dependent variable *Y*, where the effect of *X* on *M* represents the “*a* path,” and the effect of *M* on *Y* controlling for *X* represents the “*b* path.” Mediation effects may be quantified as the product of the *a* and *b* paths (MacKinnon, 2008). Additionally, the total effect of *X* on *Y* depicts the “*c* path” in the bottom portion of Fig. 1B, which is equal to the sum of direct (*c′*) and indirect effects (*ab*).

Theories and hypotheses may also posit relations involving the combination of moderation and mediation processes, often referred to as “moderated mediation^5^,” which implies “conditional indirect effects.” We loosely describe this integrated model as one where the magnitude or direction of an indirect effect depends on levels (e.g., assumed values or contexts) of a moderating variable, although it is important to note that there are multiple conceptual and analytical definitions of moderated mediation (e.g., Muller et al., 2005; Preacher et al., 2007). Figure 1C illustrates a common case of moderated mediation in which an indirect effect is made conditional as a function of *W* moderating the *a* path (e.g., Hayes model 7; Hayes, 2017). That is, the differences in the *a* path across levels of *W* produce differences in the indirect effect *ab* as well. Preacher et al. (2007) discussed this case along with four other ways *ab* could be moderated (e.g., *X* or *W* moderates the *b* path, *W* moderates both *a* and *b* paths, or *a* and *b* paths are moderated by separate moderators), but we keep our focus on *W* moderating the *a* path throughout the paper briefly noting extensions to the other four models to simplify our presentation of examples.

A classic approach to mediation used separate regression models (Baron & Kenny, 1986); however, SEM is a multivariate approach that simultaneously models multiple systems of equations, making it more ideally suited to model hypotheses involving mediation. Indirect effects in SEMs may be investigated to be conditional of a categorical variable via single-group (e.g., moderator is represented by a variable(s) in the model) and multigroup analysis (e.g., observations are segregated into groups using the levels of the moderator such that the variable is not included in the model; Ryu & Cheong, 2017). The strengths and weaknesses of each approach are discussed throughout the tutorial.

### Technical tutorial

Given the lack of intuitive resources on facilitating MC simulation power analyses with fixed covariates in simsem, we created a comprehensive repository to house all the coding syntax rather than compromise detail to make it fit in the text. Each section in this paper has a corresponding section in an R-code vignette, which can be accessed via our online appendix (https://osf.io/mpd74/). The core syntax related to the tutorial is provided in tables, but we occasionally refer to some additional material in the more comprehensive vignette. Each row of text in the vignette is numbered, which will be used to reference specific chunks of code throughout the technical discussion. Only text lines (including code outputs) are numbered, therefore chunks of code are specified in brackets denoting the row number immediately preceding the referenced code (e.g., [45] referencing the first block of code in the vignette). The exception to this formatting is when referencing R Console output, which will correspond to the exact row number. Our tutorial assumes a degree of familiarity with R basics and structural equation modeling (SEM) software; thus, those with less experience may benefit from the following resources covering R (https://swirlstats.com/students.html) and SEM (Beaujean, 2014; Rosseel, 2012) more thoroughly.

**Fig. 2:**
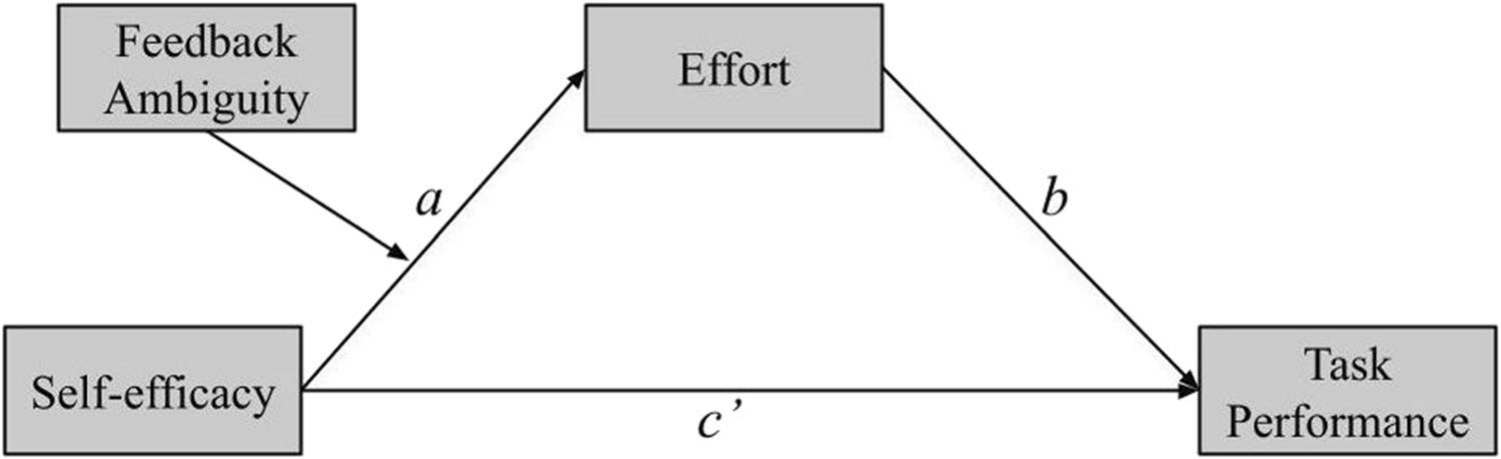
Running example of moderated mediation. *Note*. Illustrative case of moderated mediation used as a running example throughout the tutorial

To aid in the interpretability of our discussion, we introduce a running example of moderated mediation with dichotomous treatment effects in Fig. 2 below. That is, self-efficacy (control vs. treatment categorical exogenous variable) affects task performance (continuous endogenous variable) through effort (continuous endogenous variable) while performance feedback ambiguity, or “feedback ambiguity” for short (unambiguous control vs. ambiguous treatment categorical exogenous variable) moderates the *a* path and thus the indirect effect *ab*. In this example, self-efficacy (one’s belief in their capacity to execute behaviors necessary for goal attainment; Bandura, 1977) is manipulated in the treatment group by providing subjects false normative information to decrease their perception of task difficulty (increase self-efficacy). Whereas feedback ambiguity is manipulated in the treatment group by restricting subject’s feedback on performance while engaging in the task, feedback is provided continuously in the control condition.

For simplicity and pedagogical purposes, we first discuss the simple mediation version of self-efficacy affecting task performance through effort, and then build on this model in the second half of the tutorial by incorporating the moderator, feedback ambiguity. For both simple mediation and moderated mediation sections, we walk through single- and multigroup approaches. Lastly, a general simsem workflow is illustrated in Table 1, which is consistent in each of the following sections.

**Table 1:** Simsem workflow

Step	Description
1)	Specify fixed distribution of predictor(s) – in our example, an experimental design matrix, but can be any arbitrary distribution(s).
2)	For each parameter matrix, specify (1) fixed/free estimates and (2) data-generating parameter values and bind() each pair together.
3)	Collect all parameter matrices into a model().
4)	Use sim() to simulate and analyze nReps= samples of n= simulees.
5)	Summarize Monte Carlo results.

Steps outlining the simsem workflow for Monte Carlo power analysis.

## Simple mediation

Given that our exogenous variables represent assigned/manipulated groups rather than numeric values, we employ “dummy coding” to denote self-efficacy group notation. In our simple mediation model, assuming a 0 = control, 1 = treatment dummy coding pattern, the *a* path represents the difference in means of effort between the self-efficacy control and treatment groups, while the *b* path is the effect (i.e., the partial coefficient) of effort on task performance controlling for self-efficacy, which is constant across self-efficacy treatment and control groups. Lastly, the *c′* path is expressed as the adjusted mean difference in task performance between self-efficacy groups, controlling for effort.

### Specify a single-group population model

We begin by installing and loading the simsem package (http://simsem.org/) in R using standard syntax.> install.packages("simsem")> library(simsem)

In simsem, the effects of our dichotomous predictor self-efficacy can be generated according to our study design as fixed dummy-coded variables. The self-efficacy conditions are coded as 0 for control (no self-efficacy manipulation) and 1 for treatment (increased self-efficacy). We design a matrix of these dummy codes by first specifying the number of subjects to be randomly assigned per group. In our example, we arbitrarily assigned 50 participants per group (Table 2, line 1), but this is only a starting point because sample size can be adjusted later as a function of our power analysis results. After assigning the number of participants per group, we use the data.frame() function to populate a matrix comprised of a column X (self-efficacy) with 100 rows indicating which group (self-efficacy condition = 1 or 0) each observation in the data frame corresponds to (Table 2, line 2). Note that these are balanced groups, so 50 of the rows are filled with dummy code = 1 and 50 with dummy code = 0. We name this data frame containing the dummy codes of our fixed self-efficacy variable “exoData” to be integrated into our model later. Lastly, sample size is equal to the number of rows within exoData (*N* = 100), which we specify by using the nrow() function and assigning the object to “N” (Table 2, line 3). Because sample size is coded as a function of the number of rows in exoData, we only have to update the number of subjects per group when iteratively increasing/decreasing sample size to obtain desired power later on.

**Table 2:**
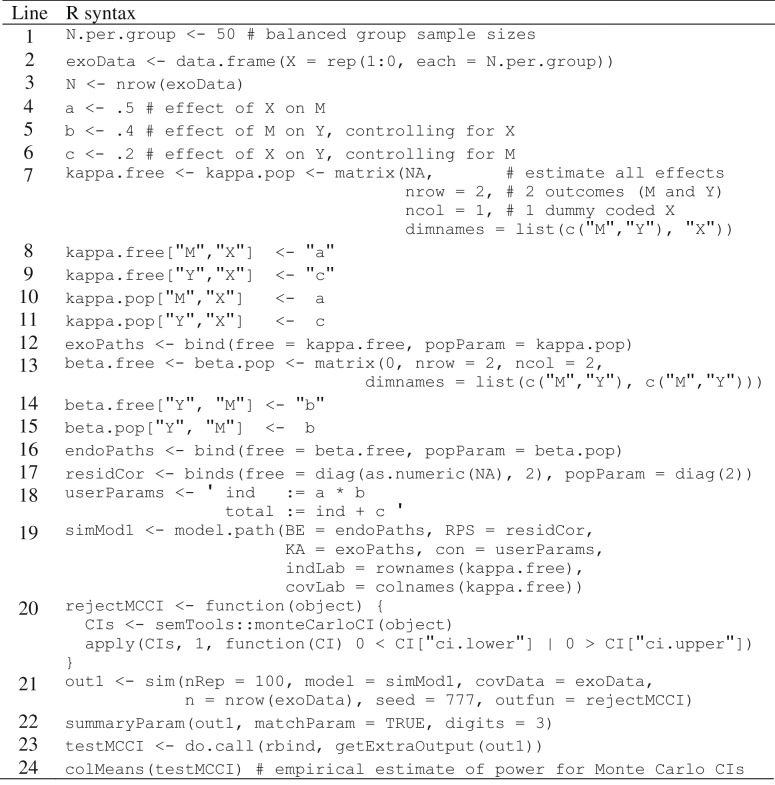
R syntax for Monte Carlo power analysis of simple-mediation model, single-group approach

Next, we define our population parameters from which we will repeatedly draw simulated samples. These parameters include the effect of self-efficacy on effort (*a* path), the effect of effort on task performance (*b* path), and the direct effect of self-efficacy on task performance controlling for effort (*c′* path). To do so, we simply assign a value for each effect to an object in R (see Table 2, lines 4–6). These effects should correspond to the smallest effect size of interest (SESOI) thought to exist in the population (see Lakens et al., 2018 and [651] for discussion on determining SESOI and drawing estimated power curves). The simsem package can accept standardized slopes, so the SESOI input for the population parameters may be in their standardized forms; however, unstandardized effect sizes can be accommodated as well. We elaborate further below on how simsem facilitates standardized parameters.

Once standardized values for the *a*, *b*, and *c′* paths have been assigned to objects, we design matrices corresponding to the exogenous and endogenous paths to be passed to simsem. This is done using LISREL-style matrices, supplemented by additional matrixes that capture the effects of exogenous predictors (X) on observed (kappa: κ; Muthén, 2002, Eq. 1) or latent (gamma: Γ; Muthén, 2002, Eq. 2) variables. This requires us to create a “kappa” matrix for exogenous paths (i.e., effects of the treatment variable on the mediator and outcome) and a “beta” matrix for endogenous paths (i.e., the effect of the mediator on the outcome). The structure of both kappa and beta matrices contains one row for each outcome and one column for each predictor. Within each kappa and beta matrix, simsem requires us to separately specify (a) which parameters are freely estimated vs. fixed to a particular value and (b) the values for population parameters when generating data. We break this process down into the following four steps: (1) design the structure of the matrix, (2) specify the matrix of free/fixed parameters, (3) specify the matrix of population parameters, and (4) integrate and store both matrices into a SimMatrix object. The subsequent two sections walk through these steps for both our exogenous parameters (kappa matrix) and endogenous parameters (beta matrix).

Beginning with creating a kappa matrix to store our exogenous parameters, we first build the structure using the matrix() function to specify the dimensions, column names, and row names (Table 2, line 7). Initially, we fill the matrix with “NA” (the missing-data code in R) as place holders in each element, followed by the number of rows and columns, which in this case is 2 and 1 respectively, corresponding to our two outcomes and one dummy-coded exogenous predictor. Note that “NA” or a character string (e.g., “b”) indicates to simsem that the parameter should be estimated freely, opposed to being fixed when a numerical value is input. Then we label the names of the dimensions using the list() function, specifying the rows first (“M”, “Y”) followed by the column (“X”). We assign this matrix to both “kappa.pop” and “kappa.free” objects, for we use this same structure in the second and third steps to populate the two matrices independently in order to be compatible with simsem. That is, we create two matrices (kappa.free and kappa.pop) with the same dimensions and labels. Second, we replace the “NA” values in the kappa.free matrix with character-string labels for the *a* path and *c′* path with “a” and “c” respectively (Table 2, lines 8–9). Using labels rather than merely “NA” will later allow us to specify user-defined parameters in lavaan syntax, such as indirect and total effects. Third, the “NA” values in the kappa.pop matrix are replaced with the actual population parameters (Table 2, lines 10–11) consisting of the standardized effects assigned above for the *a* and *c′* paths in their respective elements. Lastly, we use the bind() function in simsem to create a SimMatrix object comprised of both kappa.free and kappa.pop matrices. This new SimMatrix we assign to an object “exoPaths” (Table 2, line 10).

Next, we repeat these four steps (with minor alterations to design) to populate beta matrices for our endogenous parameter (Table 2, lines 13–16). Again, we first use the matrix() function to set the dimensions, row names, and column names; however, now we populate each element with the value 0 rather than “NA” and design a 2 (effort, task performance) × 2 (effort, task performance) matrix because most values will be fixed to zero (Table 2, line 13). The list() function is then used to label the rows (“M”, “Y”) and columns (“M”, “Y”). This matrix structure is then assigned to both “beta.free” and “beta.pop,” which we populate in the following two steps. Second, in the beta.free matrix, the 0 in row-Y, column-M is replaced with the character-string label “b”, representing our freely estimated *b* path (Table 2, line 14). Third, in the beta.pop matrix, the 0 in row-Y, column-M is replaced (Table 2, line 15) with the parameter previously assigned to object “b” (b=.4). The remaining zeros in our beta matrices are fixed values for elements M–M and Y–Y specify that our endogenous variables cannot cause themselves, and for Y–M in order to freely estimate only the effect of M on Y and not the other way around. Finally, the bind() function passes both beta.free and beta.pop matrices into a SimMatrix, which we assign to an object “endoPaths” (Table 2, line 16).

The next step in specifying the population parameters for our simple mediation model requires us to create a covariance matrix of residuals among endogenous variables (Table 2, line 17). This process is quite similar to that of step 4 in the previous section, for we again create two 2 (effort, task performance) × 2 (effort, task performance) matrices and pass them both to the bind() function. The first matrix indicates which parameters to freely estimate or fix to specific values (only variances on the diagonal), and the second specifies what population parameter values to use to generate data (optimal vector of variances). Because we specify a regression path between M and Y rather than a residual variance, we use the diag() function to easily create diagonal matrices in which off-diagonal elements are zero. The only freely estimated parameters are residual variances of effort and task performance, specified with “NA”, but now we also use the as.numeric() function so “NA” is interpreted as a (missing) numeric rather than logical value (the default). Rather than specifying population parameters for the residual variances, instead we specify that the marginal (total) variances of M and Y should be 1, using diag(2) to generate a 2 × 2 identity matrix (i.e., all zeros except for ones on the diagonal). We assign this object specifying our residual matrix to the object “residCor”. When we assemble all matrices of model parameters using the model.path()function, we will pass the residCor object to the argument RPS= (residual correlation matrix) rather than PS= (residual covariance matrix). That tells simsem to automatically choose residual variances that equal 1 minus the explained variance, which standardizes our population parameters because now the sum of explained and residual variances is equal to 1 (total variance = 1).

Next, we specify user-defined parameters using lavaan syntax (Table 2, line 18), as shown in the online mediation tutorial (https://lavaan.ugent.be/tutorial/mediation.html). User-defined parameters should be included in the model.path()function whenever users want their simulation results to include calculations of power, bias, etc., for functions of parameters, such as the indirect effect represented as the product of the *a* and *b* paths, and the total effect as the sum of the indirect (*ab*) and direct effects (*c* path). Defining a model in lavaan syntax requires all functions of parameters to be specified in a character string (i.e., within quotation marks). Note in Table 2, line 18, how we specify each function of parameters using the lavaan operator “:=”, which is assigned to the object “userParams”.

Finally, we combine all the specified elements above into a single SimSem model object using the model.path()function (Table 2, line 19). In addition to including the “endoPaths”, “residCor”, “exoPaths”, and “userParams” objects, we can also specify custom variable names using the indLab= argument for endogenous variables and covLab= for exogenous variables. Because we specified these names in our matrices of regression slopes, we can use the rownames()and colnames()functions to assign the same labels as our endogenous (effort, task performance) and exogenous (self-efficacy) variables, but we could also simply pass the variable names as a vector, such as c(“M”,”Y”). We assign the SimSem object to “simMod1,” which stores all specifications for the population model of our simple mediation example. Although it is not discussed here, in the vignette we use this simMod1 object to generate a single data set [85], which we use to illustrate how to analyze data using both single-group [83–126] and multigroup data analyses [127–191], which happens iteratively in the MC power analysis. This also serves as a demonstration comparing the single-and multigroup approaches, as described by Ryu and Cheong (2017).

#### Monte Carlo power analysis for single-group model

The sim() function in simsem automates the process of generating a sample and then fitting the model to the simulated data. This process is iterated many times in the sim() function, repeatedly fitting models to thousands of simulated samples. Power is then calculated as the proportion of samples in which the null hypothesis is rejected for each parameter estimated in the model. By default, lavaan uses an asymptotic approximation—the delta method (e.g., Oehlert, 1992; Sobel, 1982, 1986)—to calculate standard errors for functions of parameters, which may result in inflated type 1 error rates when indirect effect estimates have nonnormal sampling distributions (which is often the case; MacKinnon et al. 2002, 2004), particularly when sample sizes are relatively small (also common in behavioral sciences). MC estimates of confidence intervals (MCCI) are more robust than the delta method because only parameter estimates themselves (i.e., *a* and *b* paths) are assumed to have normal sampling distributions (Preacher & Selig, 2012). Using the semTools package (Jorgensen et al., 2021), we can easily obtain MCCIs for indirect effects by passing a fitted lavaan model to the monteCarloCI() function (e.g., [117, 191]), and test the H_0_ by checking whether the MCCI contains that value. To estimate the power using this more robust test, a custom function can be written that accepts a fitted lavaan model and returns the result of a H_0_ test (Table 2, line 20), and that custom function will be applied for each simulated sample by passing the function to the outfun= argument in the sim() function (Table 2, line 21).

The remaining information must also be passed to the sim() function (Table 2, line 21): First is the number of repeated samples to be simulated (in the vignette we specify 100 for faster computations, but 1000 to 5000 samples are recommended to ensure convergence and more robust estimates; Muthén & Muthén, 2002) via the nRep= argument, as well as the size of each sample n=, which must match the number of rows in exoData. The covariate data in exoData must also be passed to the covData= argument, along with the population parameters in the simMod1 object via model=. To ensure replicability of results, a default seed= for the random-number generator is set to 12345, but can be any integer (e.g., seed=777 in Table 2, line 21).

We assign the sim output to an object “out1” and pass it to the summaryParam() function to inspect power for each parameter (Table 2, line 22). Note that it may take several minutes for the MC simulation to complete, especially with larger sample sizes and number of samples. The output of the summaryParam() function displays 10 columns; for detailed information of each, enter “?summaryParam” into the R console to open the help page. Of immediate interest to us is the “estimate average” column, which is the average of the parameter estimates across all samples; the “power (Not equal 0)” column, which is the estimated power to reject a H_0_ of zero for each parameter (note power for the indirect effect is 57%); the “average bias” column, which is the difference between average estimates and corresponding population parameters (as a rule of thumb, “good” average bias has an absolute value less than .10); and the “coverage” column, which is the percentage of (1 – α) × 100% confidence interval covering the parameters underlying the data (by default, alpha=.05). Coverage rates that deviate substantially from nominal values (e.g., 95%) indicate the practical impact of biased point or *SE* estimates.

It is important to note that power in this output is derived from the default delta-based method, and to extract the power results from our custom MCCI function, we must pass our out1 object to the getExtraOutput() function to extract the list of sampled MCCI results stored in out1, then use rbind() to convert the list into a matrix with parameters in columns and replications in rows, which we assign to the object “testMCCI” (Table 2, line 23). We then pass the testMCCI object to the colMeans() function (Table 2, line 24) to get the average of the dummy codes indicating whether the H_0_ was rejected for the indirect and total effects. These results indicate power of 72% and 64% for the indirect and total effects respectively, which is substantially higher than the power produced using the default delta method (power for indirect effect = 57%). Since power is still less than 80% for the indirect effect, we must go back to the first few lines of code where we specify the number of subjects in each group—this value is assigned to the “N.per.group” object (Table 2, line 1). We would then iteratively increase the number of subjects per group and re-run all the code until power for the indirect effect is at an acceptable yet practical level. Keep in mind that the relationship between sample size and power is not linear; thus, it may take a large increase in sample size to obtain a small increase in power, especially in more complex models with more parameters.

### Specify a multigroup population model

Alternatively, the multigroup approach does not include our dichotomous self-efficacy predictor in the model (Ryu & Cheong, 2017). Instead, a model with endogenous variables (M and Y) is specified separately within each self-efficacy (treatment and control) group, and the effects (*b* paths) are held constant across the two models. Here, the effect of self-efficacy on effort (*a* path) is quantified as the difference in intercepts for effort between self-efficacy groups, and the direct effect of self-efficacy on task performance (the *c′* path) is the difference in intercepts for task performance controlling for effort. With the effect of effort on task performance (*b* path) and residual variances held equal across groups, the multigroup model is equivalent to the single-group model, as our online vignette illustrates using a single simulated data set.

An advantage of the multigroup approach over the single is that both of the above-mentioned constraints may be lifted. For instance, releasing the constraint on the effect of effort on task performance allows self-efficacy to moderate the *b* path (moderated mediation model 1 in Preacher et al.’s, 2007, Fig. 2; also called model 14 by Hayes, 2017) and thus to test the assumption of homogeneous slopes. Releasing the constraint on residual variances enables one to test assumptions of homoskedasticity across treatment groups. It is important to note that these benefits come with a tradeoff, for power is lost when constraints are lifted. Releasing constraints costs degrees of freedom and in turn power when using the same sample size and data because of the additional parameters required to be estimated. Conversely, if the model is too constrictive then its type 1 error rates will be inflated, which will outweigh the benefits from added power. Ideally, when employing the more flexible multigroup approach with parameters unconstrained, one could simply increase sample size to buy back power. Unfortunately, what is ideal and what is practical are often in opposition, so we provide instructions on both constrained and unconstrained multigroup approaches below.

The multigroup approach requires us to update our simsem model object to include a mean structure because the effects of self-efficacy are differences in group intercepts. To facilitate a mean structure, we can specify the population values to correspond to the distance between intercepts by creating an “alpha” vector of observed-variable intercepts. In Table 3 (Lines 1–2), we use the bind() function to generate an alpha vector for each self-efficacy (treatment and control) group. The intercepts for M and Y are allowed to be estimated freely in both groups, labeled with zeroes to indicate the control group (“a0”, “c0”) and ones the treatment group (“a1”, “c1”). For the population parameter values, another vector is specified such that the *a* and *c′* paths are cut in half (divided by 2) and subtracted from zero in the control group but added to zero in the treatment group. Thus, our population mean is zero and the differences between self-efficacy control and treatment groups’ intercepts are equal to the *a* and *c′* parameters specified in the single-group population model. Lastly, we assign our control-group alpha vector to the object “AL0” (Table 3, line 1) and the treatment group’s to the object “AL1” (Table 3, line 2). We will pass both of these vectors as a list to model.path() when creating our new two-group SimSem model object below (Table 3, line 7).

**Table 3:**
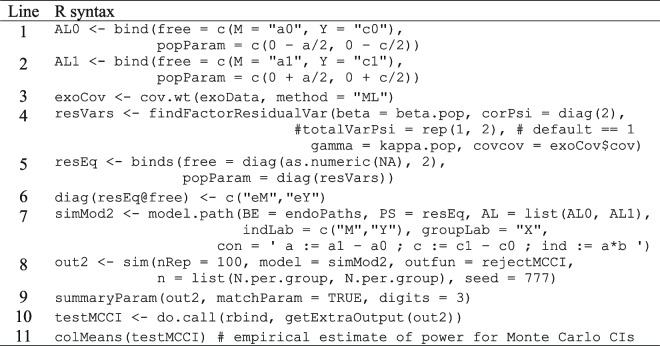
R syntax for Monte Carlo power analysis of simple-mediation model, multiple-group approach

Syntax depends on some objects created by running syntax in Table 2

Next, we generate a new matrix to specify the residual variances to be estimated freely, along with population values of residual variances for effort and task performance. Population residual variances must be specified slightly different in the multigroup model because simsem is only able to automatically calculate residual variances implied by standardized slopes within a single group (as we did in the single-group approach). To get equivalent standardizations of population parameters (i.e., in units of pooled *SD* across both groups), we can use the findFactorResidualVar() function to extract an estimate of the residual variances from a single-group model. However, we first must use the cov.wt() function to extract the population variance of self-efficacy in exoData (Table 3, line 3) to be specified as a covariance matrix among the covariates in the findFactorResidualVar() function. The cov.wt() function produces a list, which we assign to the object “exoCov” so we can extract the covariance matrix. Next, we pass arguments and objects created above to findFactorResidualVar() (Table 3, line 4) to estimate the residual variances. Note that we use the argument corPsi= to specify residual correlations and covcov= to denote our covariance matrix among covariates (self-efficacy population variance). The output is then assigned to an object “resVars”, passed to the popParam= argument when creating our matrix of residual variances (Table 3, line 5). The approach is similar to the process used for the single-group model (Table 2), except we had to specify the population variances manually rather than having simsem automate the process.

Additionally, if one wishes to constrain the residual variances across self-efficacy groups, the “NA” character string may be replaced with a new character string via the vector (“eM”, “eY”), by assigning this new character string vector to “diag(resEq@free)” (Table 3, line 6). That is, when “NA” is passed, residual variances are freely estimated for effort and task performance in each self-efficacy group, whereas specifying the same character string for effort (“eM”) and task performance (“eY”) constrains residual variances estimates to be equal across self-efficacy groups (which also adds two degrees of freedom).

With all our multigroup model specifications constructed, they can now all be passed to the model.path() function to create a new SimSem model object (Table 3, line 7). We can pass the same beta object to the BE= argument used in the single-group model (endoPaths, see Table 2); however, if we wanted to allow moderation of the *b* path as a function of self-efficacy (see Preacher et al.’s, 2007, model 1 in Fig. 2), then we would need to specify a second beta object for the second group. We pass the same resEq object to PS= (rather than RPS=). Our mean structure containing the two alpha vectors of observed-variable intercepts “AL0” and “AL1” are then passed to AL= as a list using the list() function so that each vector corresponds to their respective self-efficacy groups. Note that these vectors would not need to be passed as a list if parameters were constant across groups (e.g., no effect of X). Next, we pass a character vector indicating our endogenous variables effort and task performance (“M”, “Y”) to indLab= and a separate character vector indicating our grouping variable self-efficacy (“X”) to groupLab=. Lastly, user-defined parameters specifying our functions can be written in lavaan syntax (which now additionally includes defining the *a* and *c* paths as differences between estimated group intercepts) and passed directly to con=. This new SimSem model we assign to the object “simMod2” (Table 3, line 7).

#### Monte Carlo power analysis for multigroup model

It is important to note that the simMod1 and simMod2 models are statistically equivalent, but even with the same set.seed value, they will generate different data because the population models differ (see example data frame [275]). Thus, Monte Carlo results will differ in these two approaches, even when using the same seed for random-number generation.

Using the sim() function to conduct a power analysis using the multigroup model (Table 3, line 8) is almost identical to the process conducted for the single-group model (Table 2, line 21), with the only differences being simMod2 is passed to model=, sample size per group is specified as a list (not a vector) containing equal observations in each group (N.per.group, specified twice because groups are balanced), and we can omit the specification of the exogenous covariate data (exoData was simply used to produce population residual variances; Table 3, lines 3–4). We assign this new collection of specifications to the object “out2”, which is then passed to the summaryParam() function to produce our results (Table 3, lines 8–9). Lastly, to view the power from the MCCI test, we repeat the same procedure used for the single-group model (Table 3, lines 10–11). Note that power estimated using the default delta-based method and the MCCI method in the multigroup model produce very similar results for the indirect effect as the corresponding estimates in the single-group power analysis (power for *ab* single-group: delta-method = 57%, MCCI = 72% vs. power for *ab* multigroup: delta-method = 57%, MCCI = 71%).

## Moderated mediation

Building on our simple mediation example, we add another dichotomous variable “feedback ambiguity”, which is coded as 0 (unambiguous feedback) or 1 (ambiguous feedback), assuming that unambiguous feedback serves as the control condition, and that the primary interest is in comparing the treatment effect between unambiguous and ambiguous feedback conditions. In our example of moderated mediation, feedback ambiguity moderates the indirect effect of self-efficacy on task performance through effort (i.e., moderating the *a* path). However, there are many ways in which an indirect effect may be conditional as a function of a categorical exogenous variable, some of which do not include a fourth variable. For instance, a treatment effect in a three-variable mediation system (e.g., self-efficacy) could in fact moderate the indirect effect through the *b* path (e.g., self-efficacy moderates the effect of effort on task performance, and in turn the indirect effect on task performance through effort), which we briefly described above in the multigroup section as an example of Preacher et al.’s (2007) model 1 or Hayes’ (2017) model 14. It is infeasible to thoroughly discuss all possible moderated mediation configurations in one tutorial, such as the five common moderated mediation models [337–341] covered by Preacher et al. (2007) with dichotomous exogenous variables. In this section, we discuss Preacher et al.’s model 2 (moderation of *a* by *W*; also Hayes’, 2017, model 7), and briefly mention extensions to Preacher et al.’s models 3, 4 and 5 (see also Hayes’, 2017, models 14 and 21).

### Specify a single-group population model

Specifying population parameters for the single-group moderated mediation model is quite similar to the procedure implemented for the simple mediation model, but now we must include our fourth variable, feedback ambiguity, as well as an interaction term (self-efficacy × feedback ambiguity), such that feedback ambiguity moderates the *a* path (Preacher et al.’s, model 2). We begin by specifying the number of observations per group (Table 4, line 1), which in this model contains four groups producing a total sample size equal to the number of observations per group multiplied by four (e.g., *N* = 25 × 4 = 100). Next, we design a covariate matrix containing our dummy codes for self-efficacy and feedback ambiguity (Table 4, line 2) and add a third column containing the interaction of self-efficacy and feedback ambiguity (Table 4, line 3). When coding both self-efficacy and feedback ambiguity as dichotomous dummy codes, there are four possible dummy code combinations corresponding to each condition. After inspecting the design matrix, note how the interaction term is simply the product of the self-efficacy and feedback ambiguity dummy codes. To populate the covariate matrix with equal group sizes (Table 4, line 4), create as many copies of it as the desired number of observations per group (e.g., 25), then stack them within the same data frame and assign it to the object “exoData.”

**Table 4:**
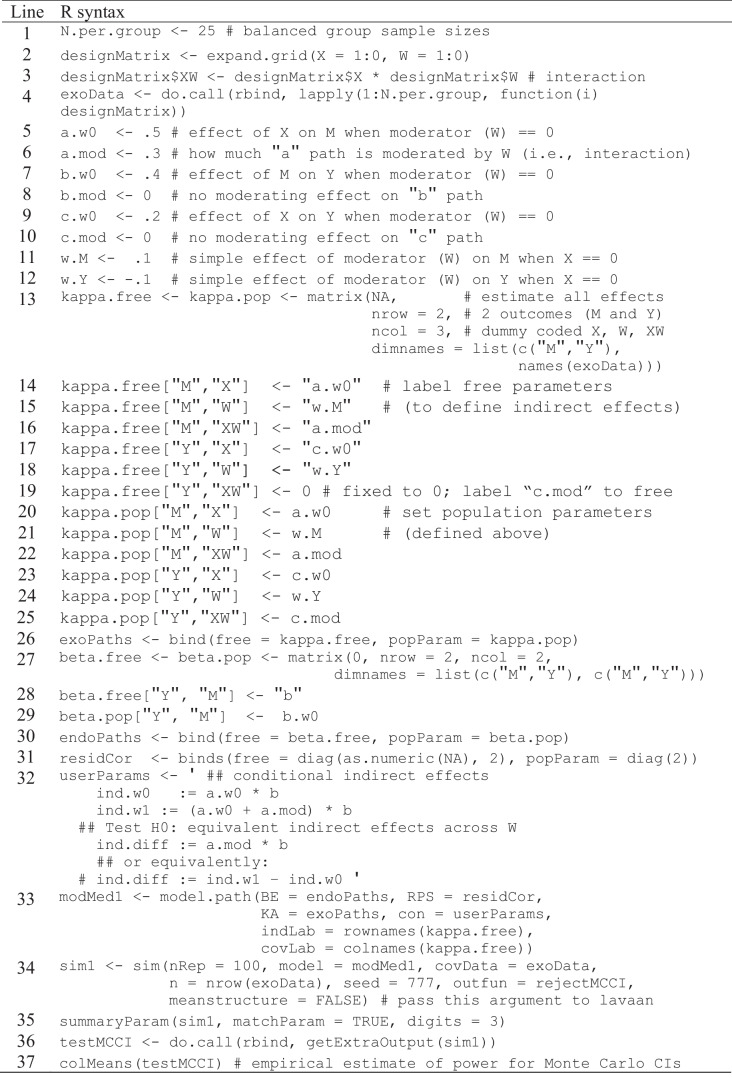
R syntax for Monte Carlo power analysis of moderated-mediation model, single-group approach

Setting population parameters for our moderated mediation model requires us to specify population values for the *a*, *b* and *c′* paths, similar to the simple mediation example, but now we must also specify the simple and moderating effects of feedback ambiguity. We must specify the effect of *X* on *M* (i.e., *a*) when feedback is unambiguous (*W* = 0) and how much the effect changes when feedback is ambiguous (*W* = 1). In this example (Table 4), we keep the simple effects (*a*, *b*, and *c′* paths) the same as in the simple mediation example (a.w0 = 0.5, b.w0 = 0.4, c.w0 = 0.2; see Table 4, lines 5, 7, and 9) and add values corresponding to effect change in ambiguous feedback (a.mod = 0.3, b.mod = 0, c.mod = 0; see Table 4, lines 6, 8, & 10). Because only the *a* path has an interaction with feedback ambiguity, we specified no change in feedback ambiguity conditions for the *b* and *c′* paths in this example. Lastly, one could specify the simple effects of feedback ambiguity on effort or task performance. Despite this effect not being of primary interest to us, arbitrary nonzero values may be set for these two parameters (Table 4, lines 11–12). Recall that these population parameters may be standardized values and correspond to the SESOI (e.g., Lakens et al., 2018).

There is currently no overarching prescription for specifying the SESOI, particularly with respect to conditional indirect effects, which veers into a complex and dynamic domain well beyond the scope of this paper. To help build a general sense of intuition and direction for researchers estimating population effect sizes in moderated mediation models with categorical exogenous variables, we briefly define the effects often needing to be estimated. The *a* and *c′* paths represent differences in means between control and treatment groups, which is often calculated via Cohen’s *d* (difference in means divided by the groups’ pooled residual standard deviation), although Cohen’s *d* is only truly defined for the two-group case (i.e., not controlling for a covariate, as in the *c’* path). Given *W* moderates the *a* path, the difference in the *a* path between treatment and control groups of *W* may be interpreted as the difference in Cohen’s *d*s representing how much *W* moderates the *a* path. Given a continuous mediator and outcome, the *b* path may be interpreted as a standardized partial regression coefficient, similar (but not equivalent) to a partial correlation between *M* and *Y* controlling for *X*. There are also many tools available for calculating *d*s vs. betas (e.g., https://www.campbellcollaboration.org/escalc/html/EffectSizeCalculator-SMD22.php). Lastly, at the end of our vignette, we provide a section “Types of Power Analysis” [651] discussing conceptualizations, approaches, and R tools for estimating various SESOI in detail.

Building on the LISREL matrices from the simple mediation examples, we employ the same four steps generating kappa and beta matrices with the addition of the exogenous variables feedback ambiguity and its interaction term with self-efficacy. Again, these steps are: (1) design the structure of the matrix, (2) specify free/fixed parameters matrix, (3) specify population values matrix, and (4) integrate and store both matrices into a SimMatrix. Beginning with the kappa matrices (Table 4, lines 13–25), we design a 2 × 3 matrix that is populated with “NA” in each cell via the matrix() function, along with the list() of dimension names for (a) the rows corresponding to our two endogenous variables via passing a character vector and (b) the columns corresponding to our three exogenous variables via pulling the column names from exoData. This data frame is then assigned to both kappa.free and kappa.pop objects (Table 4, line 13). In step 2, parameters estimated freely are specified with character strings in kappa.free, corresponding to their respective population value labels (Table 4, lines 13–19). In our example, we allow all exogenous paths to be estimated freely except for the effect of the interaction term on task performance, which we choose to fix to zero for it has no direct effect on task performance in our model. In step 3, parameter values are specified to kappa.pop (Table 4, lines 20–25). Finally, in step 4 we use the bind() function to combine both kapa matrices into one SimMatrix assigned to the object, “exoPaths” (Table 4, line 26), containing what parameters to estimate freely and the population values of each parameter.

Specifications for the endogenous paths via beta matrices (Table 4, lines 27–30) are almost unchanged from the simple mediation example (Table 2, lines 13–16), with the only differences being the population value set in beta.pop (step 3) is labeled b.w0 rather than just “b” because it is the simple effect of effort on task performance when feedback is unambiguous (control group). Of course, the *b* path is equal to 0.4 in the simple mediation example as well as in both feedback ambiguity condition groups in this moderated mediation example, so the effect of effort on task performance when feedback is ambiguous (b.w1) is not moderated; thus, the moderating effect (b.mod) is specified to be zero. Step 4, both beta matrices are combined into a SimMatrix and assigned to the object “endoPaths” (Table 4, line 30). The process of generating a covariance matrix of residuals for both endogenous variables (Table 4, line 31) is also identical to that in the simple mediation example (Table 2, line 17).

Again, we specify user-defined parameters in lavaan syntax (Table 4, line 32), which requires the addition of conditional indirect effects by first defining the indirect effect in each feedback ambiguity condition. In the vignette we assign these functions to (ind.w0) and (ind.w1) corresponding to the indirect effect when feedback is unambiguous and ambiguous, respectively. Recall that the *a* path in the treatment condition (ambiguous feedback) is equal to the sum of the *a*-path in the control group plus the change in effect in the treatment; therefore, the *a* path in ind.w1 is equal to (a.w0 + a.mod). Lastly, we specify an equivalence test of indirect effects (Mackinnon, 2008)—also known as an index of moderated mediation (Hayes, 2015)—in lavaan syntax by either (a) multiplying the interaction term (a.mod) by the *b* path or (b) taking the difference between ind.w1 and ind.w0, both approaches are statistically equivalent. We assign the set of user-defined parameters to the object “userParams” (Table 4, line 32) and pass all the newly specified elements to the model.path() function (Table 4, line 33), same as we did for the simple mediation example (Table 2, line 19), and assign this SimSem model to the object “modMed1”.

#### Analyze data using a single-group SEM

Given the paucity of guidance about using multigroup SEM to model moderated mediation (Ryu & Cheong, 2017), we precede the power analysis with single- and multigroup analyses of a single data set, generated from the population specified above. For readers unfamiliar with how to analyze moderated mediation models using single- or multigroup SEM, these examples can help clarify the MC power analyses that follow.

With our population model specified, we can demonstrate the process for generating a sample of data based on the specifications defined in modMed1 (Table 5, lines 1–2), then fit the single-group moderated mediation model defined in lavaan syntax (Table 5, line 4) to our generated data. After setting a random-number seed to ensure replicability (Table 5, line 1), we pass the sample size (i.e., number of rows in exoData object), data frame of dummy-coded exogenous variables (exoData), and our population model parameters (modMed1) to the generate() function, which we assign to the object “datmod” which contains a single simulated sample (Table 5, line 2). These data may then be fit to our model using simsem’s analyze() function using the model specifications in modMed1 (Table 5, line 3), or by specifying the model in lavaan syntax to pass to the sem() function (Table 5, lines 4–5). We pass results to the summary() function (Table 5, line 6) to inspect the estimated parameters, including the delta-method test of indirect effects, which we also test with the MCCI method in semTools (Table 5, line 7).

**Table 5:**
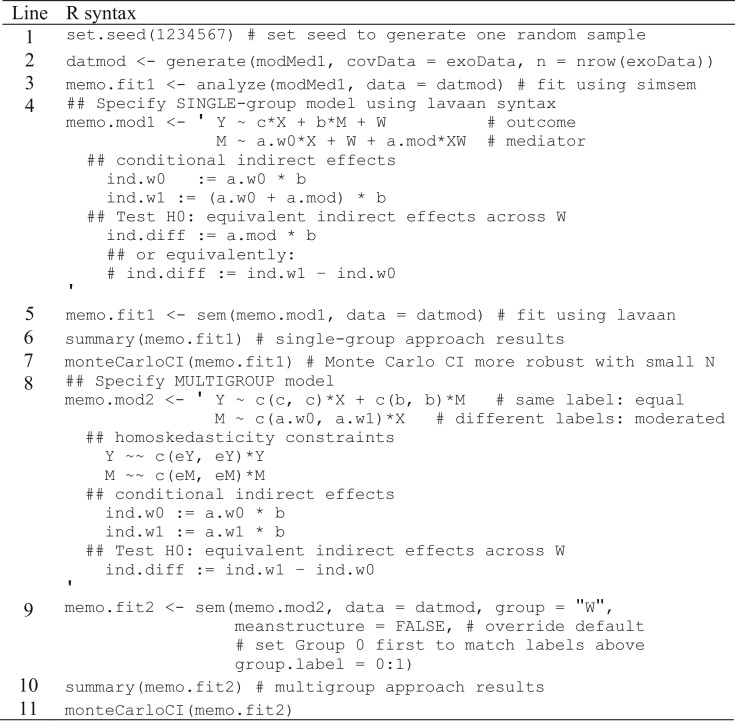
R syntax for single- and multigroup approaches to moderated-mediation analysis

Syntax depends on some objects created by running syntax in Table 4

#### Analyze data using a multigroup SEM

Building on the multigroup approach for simple mediation, linear equations are still specified separately in each group (e.g., self-efficacy conditions), but now the moderator (feedback ambiguity) is treated as a grouping variable such that each simple mediation model is defined separately for each feedback ambiguity condition. It is important to note that the example described in this section illustrates using only the moderator as a grouping variable, although the focal predictor is also a grouping variable (and treated as such in the simple-mediation multigroup example). The two-way interaction between self-efficacy and feedback ambiguity could be implemented by creating a four-group variable crossing the control and treatment conditions of self-efficacy and feedback ambiguity, but the pattern of equality constraints on intercepts to represent the two-way interaction of interest would be unnecessarily tedious, losing the advantage of intuitively interpreting moderating effects as differences between groups’ coefficients. However, if it is useful in some circumstances (e.g., to allow for heteroskedasticity across all four conditions), then we encourage future research into how this can be accomplished.

Residual variances in the multigroup moderated mediation model can also vary across groups as in the multigroup simple mediation model above, but in the multigroup moderated mediation model this accounts for heteroskedasticity across the feedback ambiguity conditions rather than across the self-efficacy conditions. Furthermore, the regression slopes for the effect of self-efficacy on effort (*a* path), effect of effort on task performance (*b* path), and effect of self-efficacy on task performance (*c′* path), can differ as a function of the feedback ambiguity condition. Constraining equal residual variances and regression slopes (e.g., *b* and *c′* paths) produces the same effect and standard error estimates as the single-group model memo.fit1 above. In contrast to the single-group model, the multigroup model has *df* > 0, which offers the advantage of testing homogeneity by releasing various combinations of constraints.

To generate data and analyze the model fit of a single sample, we specify user-defined parameters in lavaan syntax (Table 5, line 8). Parameter labels are specified in a vector (i.e., one label each for control and treatment groups). First, task performance (Y) is regressed onto self-efficacy (X) and effort (M) with equality constraints imposed by including the same labels within each vector (i.e., slopes for self-efficacy on task performance (c path) are equivalent in both control and treatment groups, and slopes for effort on task performance (b path) are equivalent in both control and treatment groups). Next effort is regressed onto self-efficacy; however, because this path is moderated by feedback ambiguity, the vector contains different labels denoting the slope of self-efficacy on effort in the control group (a.w0) and the treatment group (a.w1). Homoskedasticity constraints are then defined by specifying the residual variance of task performance in a vector with the same error labels, as well as the residual variance of effort in a vector with the same error labels. Conditional indirect effects are then defined for each level of the moderator: one for the indirect effect in the control group (a.w0 * b) and another for the indirect effect in the treatment group (a.w1 * b). Additionally, an equivalence test of indirect effects is defined as the difference between the indirect effects in each feedback ambiguity group. These user-parameters defined in lavaan syntax are assigned to the object “memo.mod2” (Table 5, line 8).

To fit our multigroup moderated mediation model (Table 5, line 9), the model syntax is passed to the sem() function, along with the same generated data “datmod” (Table 5, line 2) created for the single-group example. Additional arguments include specifying the grouping variable (group = “W”) and meanstructure=FALSE to omit the irrelevant mean structure; we also set group.label=0:1 to guarantee that the control group (0) is the first group, so the direction of user-defined parameters is as expected. We then assign this fitted model to the object “memo.fit2” and pass it to the summary() function to inspect results (Table 5, line 10). Note that regression and variance estimates are equivalent across both feedback ambiguity groups (group 1 = control, group 2 = treatment), and to the estimates produced in the single-group moderated mediation model. Releasing any combination of the four constraints on variance or slope estimates in lavaan syntax (memo.mod2) may be done to test assumptions of homoskedasticity or homogeneous slopes, respectively. Again, we pass memo.fit2 to the monteCarloCI() function to obtain robust MCCIs for user-defined parameters (indirect effects in each moderator group and difference between groups), as an alternative to lavaan’s default delta-based method (Table 5, line 11).

### Power analyses

#### Single-group simulation

MC power analysis can be carried out for the single-group moderated mediation model using the sim() function (Table 4, line 34) and passing nearly the identical arguments as in the simple mediation example (Table 2, line 21). The only differences in syntax passed to the sim() function is we now specify our single-group moderated mediation model modmed1 and pass FALSE to the argument meanstructure= (again, the number of repetitions should also be increased to 1000–5000 to ensure convergence; Muthén & Muthén, 2002). This object is then assigned to “sim1” and passed to the summaryParam() function to yield the results of the MC power analysis (Table 5, line 35). Lastly, to estimate power using the MCCI method (Table 5, lines 36–37), we “do a call” of rbind() to the list of test results returned by getExtraOutput() from our sim1 object, assigning the data frame to the object “testMCCI” and estimating power by passing testMCCI to the colMeans() function. Note in our example that power estimates derived from delta-method *Se*s (e.g., power estimate of conditional indirect effect = 9%) are lower than estimates produced via MCCI (power estimate of conditional indirect effect = 13%). The sample size may be increased or decreased iteratively in Table 5, line 1 to determine the necessary sample size (per group) for the desired power level.

#### Multigroup simulation

As mentioned in the simple-mediation multigroup section above, the multigroup approach can facilitate testing heterogeneity of variance or slopes, which would be required for simulating data from any model in Preacher et al.’s (2007) or Hayes’ (2017) taxonomy. The multigroup simple-mediation model facilitates Preacher et al.’s model 1 (X moderates *b*), and although this section focuses only on Preacher et al.’s model 2 (W moderates *a*), multigroup multiple-mediation models also facilitate Preacher et al.’s model 3 (W moderates *b* path), model 5 (W moderates both *a and b* paths), and model 4 (*a* and *b* paths are moderated by different exogenous variables; facilitated by adding an additional covariate to moderate *b*). Although it is not the focus of this article, our following example shows how the multigroup approach can be used to model heterogenous variances.

In the multigroup model, the matrix containing dummy-coded exogenous variables must include the grouping variable (feedback ambiguity; *W*), despite the grouping variable not being an explicit predictor in the model. For simsem to recognize it as a grouping variable, we must first copy the grouping variable “W” to exoData using the factor() function (Table 6, lines 1–2), so numeric codes denote categories rather than numeric values. We indicate the levels=0:1 should be assigned labels=1:2 corresponding to the two levels of feedback ambiguity (control and treatment group). A grouping variable’s values must be sequential integers starting with 1 and ending with the number of groups, which is why they receive the labels= 1:2 rather than 0:1. We do not include the interaction column (“XW”) from the exoData data frame (Table 6, line 1), ensuring that the grouping variable is the last (furthest right) column in the data frame, which is pertinent for compatibility with simsem.

**Table 6:**
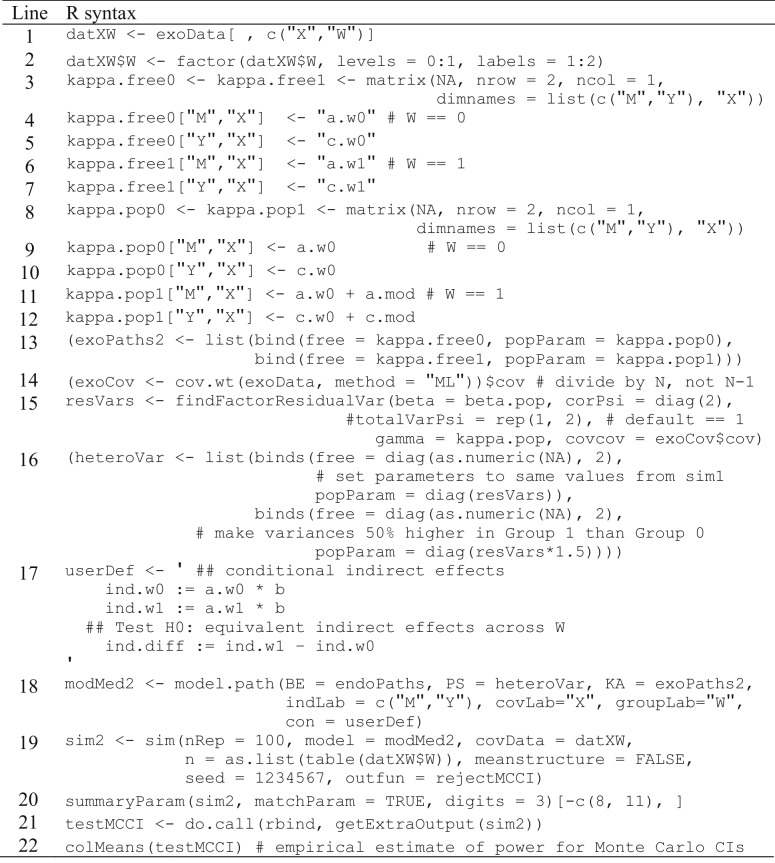
R syntax for Monte Carlo power analysis of moderated-mediation model, multigroup approach

Syntax depends on some objects created by running syntax in Table 4

Next, we must update our matrices to accommodate multigroup moderated mediation with heterogeneity. The kappa matrices in the single-group simple mediation section (Table 2, line 7)—which had the same three modeled variables (X, M, and Y)—can function as the foundation of our new matrices, with the addition of specifying kappa.free and kappa.pop for each level of feedback ambiguity and updating their labels (Table 6, lines 3–13). The beta matrices from single-group simple mediation endoPaths (Table 2, lines 13–16) can be used without any modifications because the *b* path has equivalent population values (unless the *b* path was also moderated, as in Preacher et al.’s models 3 and 5). Once kappa.free and kappa.pop matrices have been updated, a new exoPaths object may be created containing both control treatment group specifications (Table 6, line 13).

When defining residual variances, we now must specify which parameters are freely estimated and their population values in each feedback ambiguity group. The syntax for freely estimated parameters remains the same (e.g., free = diag(as.numeric(NA, 2)) as in the previous sections (Table 2, line 17; Table 3, line 5; Table 4, line 31), but now we can input the pooled population values from sim1 (e.g., popParam= diag(c(M = 0.8718434, Y = 0.8061616)); see [504, 505]). Alternatively, one could use the findFactorResidualVar() function to set total variances to 1, so that regression slopes are standardized parameters (Table 6, lines 14–15). To add heterogeneity of variances (Table 6, line 16), we make the variances in the treatment group of feedback ambiguity 50% higher than the control group by multiplying the pooled population variance values by 1.5. Again, we pass all specifications to the list() function and assign our matrix defining residual variances to the object “heteroVar”.

Next, we create user-defined parameters assigned to the “userDef” object in lavaan syntax (Table 6, line 17) by specifying the indirect effect in the control group of feedback ambiguity (ind.w0 := a.w0*b), the treatment group (ind.w1 := a.w1*b) and the difference between the two indirect effects in each group (ind.diff := ind.w1 – ind.w0). Now that all model parameters are defined, we can consolidate them using the model.path() function, specifying endoPaths as our beta matrices, heteroVar as our residual variance-covariance matrix (using PS=), exoPaths as our kappa matrices, vector c(“M”, “Y”) as our indicator labels, “X” as our character vector of covariate labels, “W” as our labeled grouping variable, and userDef as our user-defined parameters, assigned to the object “modMed2” (Table 6, line 18). Lastly, we can run the MC power analysis using the sim() function (Table 6, line 19) with arguments near identical to those in the single-group approach above (Table 4, line 34). The two differences being (a) modMed2 is specified as our model= and (b) the vector of group sample sizes must be passed as a list and reflect the number of observations in each *W* group. To input the number of observations in the exogenous variables, we simply used the table() function to count the number of rows per level of *W* in our covariate data frame, converted to a list so sim() understands it is the sample size per group (i.e., a vector of sample sizes would be interpreted as the *N* used per replication). These arguments are assigned to the object “sim2” and passed to the summaryParam() function (Table 6, line 20) to display the power estimates using delta-method *Se*s. The estimated power of the delta method is 5% for our conditional indirect effect [589], whereas the MCCI approach (Table 6, line 21–22) produced a power estimate of 10% [608]. As in the single-group approach to moderated mediation, MCCI had greater power estimates than the delta method.

## Conclusions

In this paper, we outlined the various challenges associated with sample-size planning for studies that test moderated-mediation models, with a specific focus on how the consideration of categorical exogenous predictors and moderators affects this process. We outline a set of tools that allow researchers to optimally plan for appropriate sample sizes to test such models. Given the increasing popularity of moderated mediation models—as represented both in theory and in primary empirical studies that test such models—considering ways of optimizing tests of such effects is critical. This is particularly important, given increasing criticism of these models in the literature (e.g., Rohrer et al., 2021), specifically with respect to their statistical power (Montoya et al., 2021). Although we have mentioned other ways to model moderated effects, the methods discussed here can also be extended to model multiple (parallel or serial) mediators, as discussed in the tutorial for Monte Carlo power analysis by Schoemann et al. (2017). Our hope is that the “toolkit” presented here will inspire researchers to more carefully consider the sample-size requirements necessary to test moderated mediation models and adopt a more critical perspective on the strengths and limitations of such models.

## Footnotes

1 Fixed covariates need not be randomly assigned treatment groups. Fixed covariates can follow from stratified sampling techniques (e.g., sampling from chosen age groups) or from characteristics of populations that arise in practice (e.g., employees with fixed distributions of qualities determined by tests administered as part of a hiring process). The single-group methods in this paper apply generally to any case when fixed covariates are relevant, but we focus on an example of experimental design because it also enables comparing single- and multigroup SEM.

2 Links to online inquiries from Research gate: https://www.researchgate.net/post/How_to_do_a_moderated_mediation_with_categorical_IV_and_a_categorical_moderator ; lavaan forum: https://groups.google.com/g/lavaan/c/otcOcc7Rclw/m/4xwhCODyAwAJ ; Web Power: https://webpower.psychstat.org/qanda/55/sample-size-for-a-3-way-moderation-and-moderated-mediation ; Web Power: https://webpower.psychstat.org/qanda/57/representing-interaction-terms-in-montecarlo-sem-power.

3 Many programming languages are capable of MC simulations with fixed covariates, however only simsem provides user-friendly tools automating some of the more difficult details of programming such MC studies.

4 A third more computationally efficient approach derived from analytical power analysis of the likelihood ratio test statistic (Satorra & Saris, 1985) is not discussed in the manuscript but is comprehensively described in the OSF appendix linked below.

5 “Mediated moderation” is another term for models that combine both features, but this is not conceptually or statistically distinct from moderated mediation (Preacher et al., 2007). For example, the term “moderated mediation” implies the focus is on the indirect effect (ab) of X on Y via M, and how ab is moderated by W. In contrast, the term “mediated moderation” implies the focus is on the moderating effect of W (e.g., the statistical interaction effect, or slope of the product XW) and how that slope is mediated by M. The same model can be used to focus on either aspect of the model. We only refer to “moderated mediation” throughout, focusing on conditional indirect effects.
